# Exploring the Impact of Telerehabilitation on Physical Performance in Patients with Pulmonary Fibrosis

**DOI:** 10.1177/26924366251377063

**Published:** 2025-09-15

**Authors:** Matti Okker, Kirsi Lindgren, Herkko Ryynänen, Marjukka Myllärniemi, Mari Ainola, Maria Hollmen

**Affiliations:** ^1^ Individualized Drug Therapy Research Program, Faculty of Medicine, University of Helsinki, Helsinki, Finland.; ^2^ Department of Pulmonary Medicine, Heart and Lung Centre, Helsinki University Hospital, Helsinki, Finland.

**Keywords:** digital rehabilitation, interstitial lung disease, quality of life, 6-minute walk test, sit-to-stand test, telerehabilitation

## Abstract

**Background:**

Interstitial lung disease (ILD) is a heterogeneous group of lung parenchymal diseases. Idiopathic pulmonary fibrosis (IPF) and nonspecific interstitial pneumonia (NSIP) are typical idiopathic interstitial pneumonias associated with significant morbidity, mortality, and reduced quality of life. Exercise training is effective for patients with various ILDs. Telerehabilitation is effective for chronic conditions and feasible in Finland via the Health Village website. We aimed to introduce the Health Village’s telerehabilitation protocol for patients with IPF or NSIP.

**Methods:**

We created a digital care pathway for patients with ILD on the Health Village website. It includes lectures, tasks, instructional videos, and guidance sessions conducted by five domain experts: a pulmonologist, physiotherapist, nutrition therapist, palliative care nurse, and social worker. We randomly selected 20 patients with ILD from Helsinki University Hospital to test the digital pathway. Each participant underwent telerehabilitation for approximately 6 months.

**Results:**

Statistically significant changes were observed in variables evaluating exercise and functional capacity. The average improvement in the 6-min walking distance was 30 m (*p* = 0.004). The 1-min sit-to-stand test showed an average increase of five repetitions (*p* < 0.001). No statistically significant differences were found in the other measures of functional capacity or quality of life. The feedback highlighted satisfaction with the telerehabilitation.

**Conclusions:**

Telerehabilitation through the Health Village pathway improved exercise and functional capacity in ILD patients, indicating its feasibility as an alternative to conventional rehabilitation. With notable physical performance improvements, telerehabilitation is a practical addition to conventional care and a viable alternative to in-person rehabilitation.

## Introduction

Interstitial lung diseases (ILDs) include lung parenchymal diseases of various origins.^1,2^ Their prevalence ranges 6.3–71 cases per 100,000 people.^3^ Idiopathic pulmonary fibrosis (IPF) is the most common and extensively researched ILD, accounting for approximately 20% of all ILD cases.^4^ Nonspecific interstitial pneumonia (NSIP), another common ILD, can occur idiopathically or in association with conditions such as rheumatoid arthritis. IPF typically has a poor prognosis, with a median survival of 2–3 years in the absence of antifibrotic therapy.^5–7^ In contrast, the 5-year mortality rate of patients with NSIP is estimated to be under 18%.^8,9^

Exercise training is effective in patients with various ILDs.^10–12^ Pulmonary rehabilitation is considered safe and has been shown to reduce dyspnea, increase exercise capacity, and improve quality of life (QoL).^11–14^ However, traditional pulmonary rehabilitation programs^15^ are not suitable for all patients. For example, frequent travel to a hospital can be challenging, and visiting hospitals may increase the risk of acquiring infections, which is a significant concern for patients with compromised lung function. Fixed schedules and sessions during business hours may not align with patients’ commitments and may limit the involvement of relatives in the rehabilitation process.

Telerehabilitation may increase the accessibility of rehabilitation services and provide the opportunity regardless of geographical location, making participation more manageable and individualized. In addition to being easily accessible, remote sessions are more cost-effective than traditional methods, particularly when considering travel expenses and time. In Finland, remote appointments have been implemented via video connections in distant areas; however, scientific research in this field is limited. Nevertheless, the limited available results have been encouraging.

Telerehabilitation utilizes telecommunication techniques to deliver therapeutic interventions, education, and counseling, as well as for monitoring and evaluating the rehabilitation process and outcomes. The field of telerehabilitation is rapidly developing.^16,17^ Studies have shown that telerehabilitation is at least as effective as inpatient rehabilitation in managing several chronic conditions.^18^ In Finland, telerehabilitation can be executed via the Health Village website, which provides a multiprofessional platform for patients and health care workers. It includes educational videos, chats, disease monitoring, self-care programs, and treatment instructions,^19,20^ contributing to address some of the problems associated with inpatient rehabilitation described above.

The goal of this study was to create a telerehabilitation program in the Health Village platform for patients with ILD, with the objective of improving the patients’ physical capacity and QoL. We also assessed its effect on exercise capacity in a pilot population of patients with ILD.

## Methods

This is a pilot and feasibility study aiming at the development and integration of telerehabilitation into patient care. The research adhered to the ethical guidelines set by the Finnish National Board on Research Integrity. It complied with the European Union General Data Protection Regulation for practical research. The study received a research permit (HUS/237/2021) and was approved by the Ethics Committee of the Hospital District of Helsinki and Uusimaa (HUS/1906/2020). All participants provided written informed consent. The study was conducted in accordance with the 1975 Declaration of Helsinki.

### Health village

The Health Village is an online platform developed by five university hospitals in Finland. Health care professionals can construct personalized self-care programs, treatment regimens, coaching modules, and therapeutic pathways. The platform aims to complement conventional hospital-based regimens with online programs. Patients are referred to specific programs, with guidance and support provided through messages from health care professionals.^19,20^

### Creating the ILD rehabilitation path

The content of the Health Village ILD rehabilitation pathway was developed by five experts: a pulmonologist, physiotherapist, nutrition therapist, palliative care nurse, and social worker.

The digital care pathway had a phased structure, allowing participants to progress after completing all materials and questionnaires from the previous phase. Each next section became available to all participants simultaneously, improving synchronicity and facilitating understanding of one aspect at a time. Participants accessed Healthy Village using a secure authentication pathway.

### Sections of the ILD rehabilitation path

The rehabilitation pathway was divided into five domains, each corresponding to a specific area of expertise. In the physiotherapy section, the focus was on the significance of exercise and physical activity with practical training instructions and examples of weekly programs. Exercise training was based on general physical activity recommendations and additional tailored information for individuals with respiratory disease. Participants selected an appropriate training program based on their preferences, background, and fitness level (light, moderate, and heavy). It was possible to change the level of the program during the rehabilitation. Exercises were performed daily for 20–60 min, depending on the weekly program intensity. Strength training instructions were delivered via videos and PDF files (links to Finnish language videos: https://www.youtube.com/watch?v=jZhjvep5n-0, https://www.youtube.com/watch?v=D-vfNyKafQ8, https://www.youtube.com/watch?v=eqxLgHZiZpw.)

Exercises were performed while standing or sitting. Participants were instructed to engage in strength training at least twice a week. Each training session included 6–8 exercises, with 10–15 repetitions per set and 2–3 sets per exercise. Exercises utilized body weight, light dumbbells, chairs, walls, towels, and resistance bands. Additionally, a light warm-up and mobility exercises were included in each session.

Physical activity was recorded in an exercise diary. Participants were instructed to record the type and form of exercise or sport, duration of activity, level of intensity, number of steps, the presence of exercise companions, and how it made them feel. Reasons for inactivity were also documented. The exercise diary was filled out online, either through the website or the mobile application. Duration, type of sport, number of steps, and reasons for inactivity were presented as open-ended questions, whereas type of exercise, presence of an exercise companion, and self-reported intensity were assessed using multiple-choice questions.

In the pulmonary fibrosis section, information on diagnosis and monitoring, prognosis, and treatment was provided.

The nutrition section covered healthy eating and self-monitoring strategies. A healthy diet included a plant-based eating plan, incorporation of healthy fats, and consumption of dietary fiber. Additional dietary instructions were provided to alleviate the side effects of pulmonary fibrosis medications.

The mental well-being section addressed coping with difficult situations, emotional resilience, values, adaptation, proactive planning, the role of loved ones, and self-care strategies. Materials included guides, videos, and writing exercises to encourage reflection and self-awareness.

In the section addressing social security, participants were provided with information on services, patient rights, and benefits. It also included video materials, text content, and essential links.

### Assessing the rehabilitation pathway

A pilot study involving 20 patients was conducted to test and validate the rehabilitation pathway. A pulmonologist specializing in ILD reviewed the electronic patient records. The inclusion criteria were (1) age ≥18 years, (2) IPF/NSIP diagnosis according to the American Thoracic Society/European Respiratory Society diagnostic criteria,^21,22^ (3) a stable disease diagnosed at least 6 months prior, (4) forced expiratory volume in 1 sec (FEV1) ≥30%, (5) diffusion capacity of the lung for carbon monoxide (DLCO) ≥30%, (6) necessary technical equipment and skills to engage in telerehabilitation, (7) adequate functional capacity for baseline and end-point measurements, and (8) medication for fibrotic lung disease had to be stable and administered at a consistent dose for at least 6 months prior to participation. The exclusion criteria included: (1) a life expectancy of ≤1 year, (2) concurrent participation in clinical drug trials, (3) severe coexisting or poorly managed conditions, and (4) previous lung rehabilitation.

This pilot study was conducted between September 2020 and April 2021. Before initiating rehabilitation, the participants underwent an initial assessment, including pulmonary function tests (forced vital capacity, FEV1, and DLCO), a 6-min walk test (6MWT), and a 1-min sit-to-stand test. Shortness of breath was assessed using the Modified Medical Research Council dyspnea scale.^23^ QoL was analyzed using 36-item Short Form Survey for health-related quality of life (an eight-dimensional health-related QoL measure). The cough visual analog scale was used to assess cough severity. The same test battery and questionnaires were used for final measurements.

Participants were asked to provide feedback on the pathway, but no structured interview was used.

### Statistical analyses

Statistical analyses were conducted using IBM SPSS Statistics, version 25 (SPSS Inc., Chicago, IL, USA). Data are expressed as means and standard deviation (SD) or as absolute numbers with percentages. All analyzed variables were tested for distribution. The independent samples *t-*test was used for normally distributed variables, and the Mann–Whitney U test was used for variables with skewed distribution. *p*-Values < 0.05 were considered statistically significant.

## Results

**FIG. 1.**
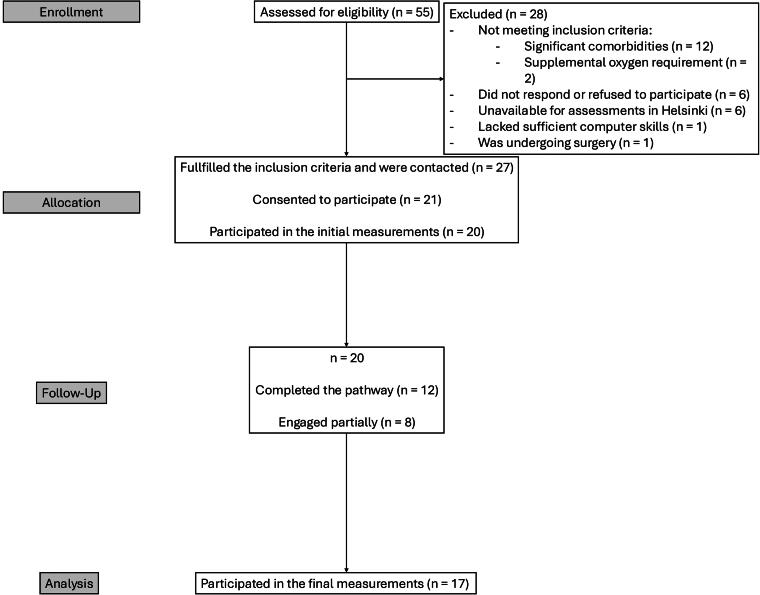
The patient selection flowchart. The graph displays patients’ enrollment, allocation, follow-up, and analysis.

Of the 55 patients included in the study, 12 were excluded due to significant comorbidities (e.g., acute coronary heart disease, cardiac insufficiency, active cancer, or mobility-limiting conditions such as arthrosis, back problems, or requiring support to mobilize). Two patients who required supplemental oxygen were excluded from this study. The pulmonologist referred 41 eligible patients. Four did not respond despite repeated contact, two refused to participate, six were unavailable for assessments in Helsinki, one was undergoing surgery, and one lacked sufficient computer skills. In total, 27 patients fulfilled the inclusion criteria and were contacted by the study nurse. They received an email invitation for signing up the rehabilitation platform. Twenty-one patients consented to participate in the study, of which 20 participated in the initial measurements. The patient selection flowchart is shown in Figure 1.

**FIG. 2.**
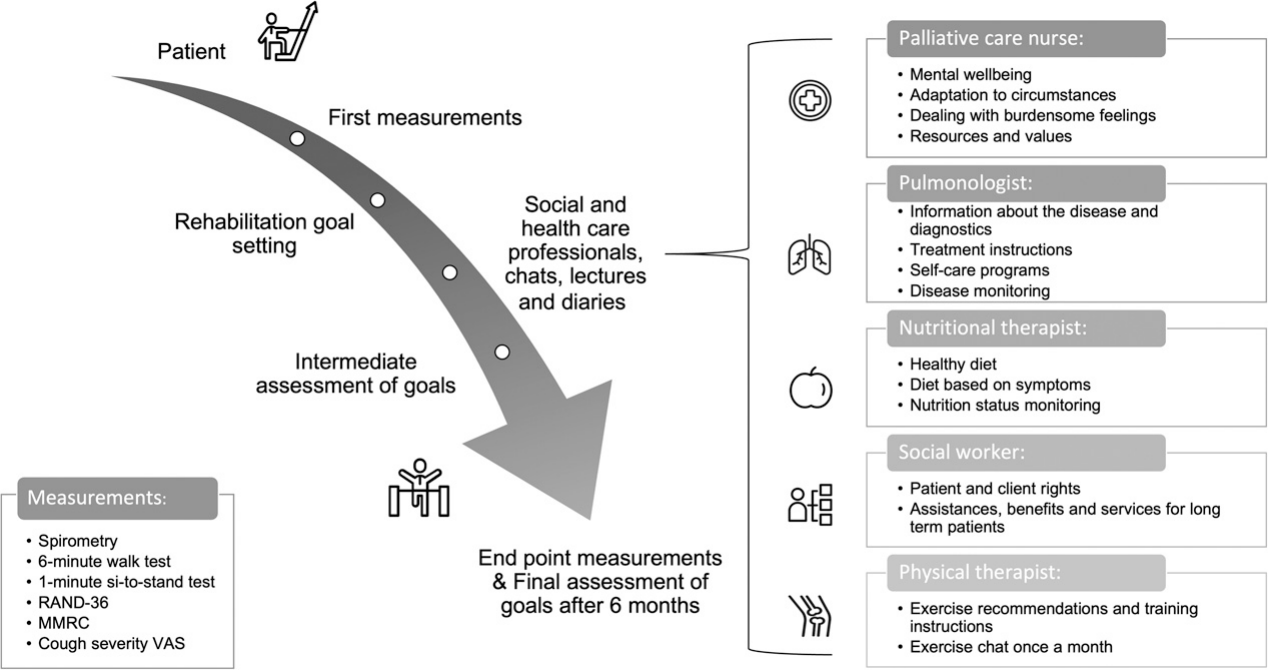
The Health Village pulmonary fibrosis rehabilitation pathway. The arrow outlines the sequence of interventions and assessments, with measurements on the left and expert domains on the right. RAND-36, 36-item Short Form Survey for health-related quality of life; MMRC, Modified Medical Research Council dyspnea scale; VAS, visual analog scale.

The Health Village pulmonary fibrosis rehabilitation pathway is illustrated in Figure 2. The materials were released step-by-step every 2 weeks, spanning 12–14 weeks. Altogether, the pathway was open for 6 months for each participant.

The demographics of the pilot study participants are presented in Table 1. Eight participants had IPF, and 12 had NSIP.

**Table 1.** Patient demographics

	All, *N* = 20
Background and comorbidities:	
Female sex	9 (45%)
Male sex	11 (55%)
Age, years	68,7
DG, IPF/NSIP	8 (40%)/12 (60%)
Smoking history,	9 (45%)
Time from quitting, years (SD)	13.36 (13.27)
Smoking years (SD)	29.14 (15.14)
BMI, kg/m^2^ (SD)	27.7 (3.14)
Comorbidities	13 (65%)
Time from diagnosis, years (SD)	5.7 (5.14)
Treatment:	
Corticosteroids (oral)	8 (40%)
Corticosteroids (inhalation)	3 (15%)
Nintedanib	3 (15%)
Pirfenidone	2 (10%)
Methotrexate	1 (5%)
Rituximab	1 (5%)
Sulfasalazine	1 (5%)
Mycophenolic acid	1 (5%)
Hydroxychloroquine	1 (5%)

Data are in *n* (%) or years. The dataset included data on background and comorbidities for all patients. However, the smoking history remained unclear for some patients.

BMI, body mass index; DG, diagnosis; IPF, idiopathic pulmonary fibrosis; NSIP, nonspecific interstitial pneumonia; SD, standard deviation.

### Exercise and functional capacity

Of the 20 study participants, 12 completed the pathway and 8 engaged partially. Seventeen participated in the final measurements regardless of how far they had progressed through the digital care pathway. One rehabilitant could not be reached despite several contact attempts, and two declined due to poor health.

**FIG. 3.**
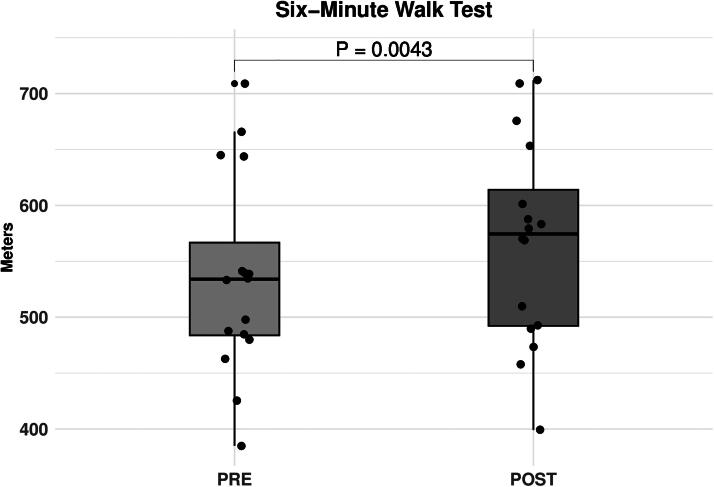
Improvement in 6-min walk test distances. The graph displays the pre- and post-intervention distances in the 6-min walk test.

**FIG. 4.**
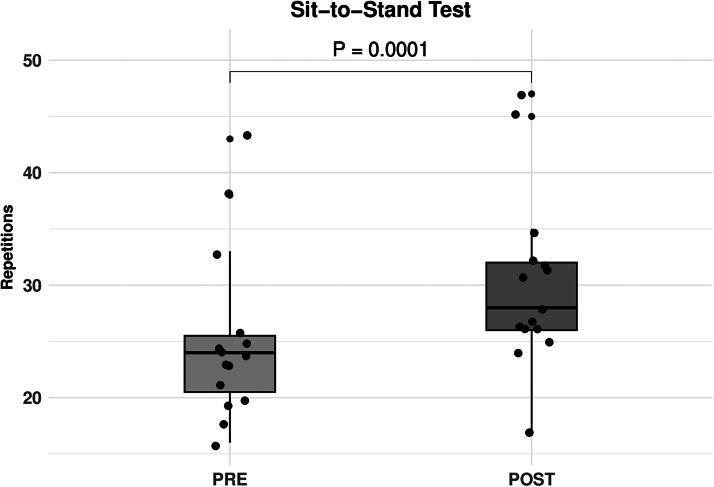
Improvement in the sit-to-stand test performance. The graph illustrates the pre- and post-intervention results of the sit-to-stand test.

Table 2 presents the pre- and post-rehabilitation results. The 6MWT distance was significantly better post-rehabilitation (mean 566.4 m) compared with pre-rehabilitation (mean 536.0 m, *p* = 0.004; Fig. 3). The 1-min sit-to-stand test results significantly improved after rehabilitation (mean 30.13 repetitions) compared with pre-rehabilitation (mean 25.13 repetitions, *p* < 0.001; Fig. 4). In patients with IPF, the 6MWT results improved by an average of 17.5 m, which was not statistically significant (*p* = 0.256). In patients with NSIP, an average improvement of 38.2 m was observed (*p* = 0.010). In the 1-min sit-to-stand test, patients with IPF improved by an average of 7.3 repetitions (*p* = 0.002), while those with NSIP improved by 3.4 repetitions (*p* = 0.019), all of which were statistically significant. There were no significant differences in dyspnea and QoL before and after telerehabilitation.

**Table 2.** Pre- and post-rehabilitation results

	Pre	Post	Change	Significance
Pulmonary function:				
FEV1, L (SD)	2.36 (0.72)	2.37 (0.72)	0.01	0.823
FVC, L, (SD)	3.09 (0.98)	3.11 (0.98)	0.02	0.545
DLCO, %, (SD)	53 (14.11)	—	—	—
O_2_ saturation before 6MWT, % (SD)	97.3 (1.34)	96.6 (1.97)	−0.7	0.119
O_2_ saturation decrease after 6MWT, % (SD)	−7.44, (5.27)	−8.94 (6.95)	−1.5	0.248
Functional status tests and symptoms:				
6-Minute Walk Test, meters (SD)	536.0 (89.6)	566.4 (91.7)	30.4	0.0043^*^
Sit-to-Stand test, repetitions (SD)	25.13 (7.44)	30.13 (7.75)	5.00	0.0001^*^
BMI, kg/m^2^ (SD)	26.7 (3.14)	27.4 (3.12)	0.7	0.833
Cough VAS (SD)	13.73 (12.08)	10.82 (9.75)	−2.91	0.464
MMRC (SD)	1.09 (0.70)	1.27 (0.90)	0.18	0.341
RAND 36:				
Physical functioning (SD)	69.55 (23.29)	69.09 (26.54)	−0.46	0.914
Role physical (SD)	59.09 (39.17)	50.00 (41.83)	−9.09	0.397
Role emotional (SD)	91.00 (15.41)	81.82 (34.56)	−9.18	0.460
Social functioning (SD)	72.91 (27.12)	78.64 (20.92)	5.73	0.513
Bodily pain (SD)	75.40 (21.37)	76.40 (17.8)	1.00	0.897
Mental health (SD)	81.80 (11.25)	79.40 (22.35)	−2,40	0.796
Vitality (SD)	66.82 (19.01)	65.45 (16.95)	−1.37	0.740
General health (SD)	50.45 (21.62)	48.00 (23.17)	−2.45	0.612

Data are presented as mean (standard deviation).

^*^ *p-*values <0.05. The data pertaining to pulmonary function, RAND 36, and functional status tests were limited to those patients who participated in both pre- and post-measurement assessments.

6MWT, 6-min walk test; DLCO, diffusion capacity of the lung for carbon monoxide; FEV1, forced expiratory volume in 1 sec; FVC, forced vital capacity; MMRC, Modified Medical Research Council dyspnea scale; O_2_, oxygen; VAS, visual analog scale; RAND 36, health-related quality of life survey instrument.

### Exercise diary

Seventeen participants recorded entries in the exercise diary, while three participants did not record any sessions. The number of recorded sessions per participant ranged from 3 to 240, with a mean of 93. The results are presented in Table 3. Step count was reported in 27.6% of sessions. Daily physical activity and endurance training were reported most often, while strength training was less frequent. Physical activity intensity was mostly light or moderate and rarely vigorous. The majority of participants exercised either alone or with a companion, while group exercise was the least common. A wide variety of sports were reported. Walking was the most common form of exercise, 47.0% of all reported sessions with a mean duration of 64.6 min (range 15–300 min, SD 33.3 min). Cycling was the second most popular sport, 6.6% with a mean duration of 58.9 min (range 30–150 min, SD 24.5 min). The rest of the reported forms of exercise were only mentioned a few times. The most common daily activities were gardening, yard work, and various household chores.

**Table 3.** Summary of exercise diary data

	Sessions per participant, *n*, (range)	Mean duration per session, min (range)	Total duration per participant, min (range)
Exercise:			
Daily physical activity	44.35 (1–122)	116.75 (20–600)	3530.24 (40–17185)
Endurance training	41.76 (0–125)	66.67 (0–192.73)	2975.71 (0–10055)
Strength training	6.82 (0–39)	27.89 (0–66.67)	240.35 (0–1121)
Light intensity	32.24 (0–98)	77.29 (0–247)	2480.06 (0–13180)
Moderate intensity	56.59 (0–147)	64.96 (0–175)	3778.06 (0–12845)
Vigorous intensity	4.12 (0–23)	88.87 (0–420)	488.18 (0–3270)
Alone	51.00 (1–132)	72.14 (10–247)	3395.06 (10–15940)
With a companion	37.18 (0–115)	90.06 (0–263.33)	2917.24 (0–11220)
Group exercise	4.76 (0–27)	46.85 (0–159.38)	434 (0–2550)
Exercise in total	92.94 (3–240)	84.47 (36.28–247)	6746.29 (140–27220)

Data are presented as means (ranges) of individual participants’ average exercise session frequencies or durations. The means were first calculated for each participant, after which group-level means were derived. The dataset was derived from entries in participants’ exercise diaries. Sessions with durations exceeding 24 h were excluded, as these were considered likely due to data entry errors.

### Participant feedback

Participants reported that the digital care pathway increased their sense of safety and support. One noted: “I felt safe and cared for.” The content was described as motivating and appropriate for the participants’ current needs: “All possible information and support are important especially at this stage!” Several participants highlighted how the pathway helped them understand their condition and how to communicate about it: “With this pathway, I’ve been able to tell my loved ones what this disease is and how I can affect the course of the disease.” Importantly, the participants also mentioned gaining reassurance about physical activity: “It turns out it’s not dangerous to get out of breath while exercising.” Some reported improvements in QoL and appreciated the peer support received through the chat feature. Comments were gathered through open-ended feedback, as no structured interview protocol was used.

## Discussion

This study evaluated the feasibility and effectiveness of a pulmonary fibrosis telerehabilitation program using the Health Village platform. We found that telerehabilitation significantly improves exercise and functional capacity in patients with ILD. To the best of our knowledge, this is the only publication to date examining the association between telerehabilitation, the 6MWT, and 1-min sit-to-stand test, along with QoL assessment. Hence, this study offers a unique contribution to the current evidence on remote functional assessment in rehabilitation.

Pulmonary rehabilitation has been shown to substantially enhance exercise capacity and QoL in individuals with ILD.^10–14^ The outcomes of our study corroborate the findings of earlier research utilizing digital platforms specifically targeting patients with IPF.^15,24,25^ The lack of significant improvement in other functional capacity measures than the 6MWT and 1-minute sit-to-stand test may reflect the limited statistical power of the study, likely due to the small sample size and relatively short intervention period. Positive impacts of rehabilitation and telerehabilitation on QoL have been documented in patients with conditions other than pulmonary fibrosis, such as asthma and stroke.^14,26^ QoL has multifactorial aspects, and in a progressive condition with a poor prognosis and significant symptom burden, it is expected to decline over time. Thus, maintaining QoL is a goal in itself, and the progressive decline in QoL over time may explain the absence of measurable improvement.^27^

The significant improvements in the 6MWT and sit-to-stand test suggest that telerehabilitation can enhance physical performance in ILD patients. The average improvement of 30 m in the 6MWT and five repetitions in the sit-to-stand test are clinically meaningful, especially considering the progressive nature of ILDs. These results reinforce prior evidence supporting the effectiveness of telerehabilitation.^12,14,24,26,28–30^ In contrast, some studies reported no 6MWT improvement with telerehabilitation,^25,31^ particularly among patients with low adherence, small sample sizes, and wide age variability. Those who discontinued rehabilitation showed a decline in 6MWT performance.^31^ No positive effect on dyspnea was detected in our study, a finding that aligns with the progressive nature of dyspnea in patients with IPF and its challenging management.^25,26^

The positive feedback from participants highlights the perceived value of the digital care pathway in providing support, education, and reassurance. Many participants reported feeling safer and more informed about their condition, which may have contributed to their engagement and adherence. The peer support facilitated through the chat feature also appears to be a valuable component, offering emotional support and shared experiences. Enhanced knowledge empowered participants to influence the disease trajectory and facilitated communication with relatives. Awareness of progress monitoring rendered rehabilitation efforts more meaningful, consistent with previous findings.^32^ Moreover, the focus on patient-centeredness in telerehabilitation is considered advantageous for individuals with chronic illnesses.^33^ Web platforms can aid in the self-management of patients with chronic lung diseases.^28^

Our study underscores the feasibility of implementing a telerehabilitation program through the Health Village platform. The high completion rate of the digital care pathway and positive feedback from participants highlight the acceptability of this approach. The participants appreciated the flexibility and accessibility of the program, which allowed them to engage in rehabilitation activities at their convenience, overcoming common barriers associated with traditional rehabilitation, such as mobility challenges, travel difficulties, and fixed schedules.^26^ The patients reported an increased feeling of safety, support, and a better understanding of their condition. Telerehabilitation offers a promising solution to increase the accessibility of rehabilitation services, especially for patients living in remote areas or those with mobility limitations. It increases the patient’s sense of security, knowledge, and even commitment to treatment. The cost-effectiveness of telerehabilitation, due to reduced travel expenses, time, and the possibility to offer rehabilitation to a larger patient population, further supports its integration into routine care for patients with ILD.^17^ Additionally, reduced need for travel also has a significant environmental impact.

The Health Village Path for Pulmonary Fibrosis was initiated in the clinic in 2023. Unfortunately, we were able to recruit only some patients before it closed for competitive tendering. As soon as the testing and updating are done, the path will reopen and provide nationwide telerehabilitation for patients with ILD.

This study has several strengths. We established a telerehabilitation protocol within the Health Village platform, offering an innovative approach to managing pulmonary fibrosis and enhancing patient engagement. The study employed a multidisciplinary approach, integrating pulmonologists, physiotherapists, nutrition therapists, and other experts, which contributed to its comprehensive and patient-centered nature.

This study had also several limitations. It was a small, single-center study. The lack of a control group, limited sample size, lack of structured interview/questionnaire regarding feedback, lack of depression and anxiety assessment with questionnaires, and short intervention duration restrict the generalizability of the findings. Self-reported exercise diaries may have introduced reporting bias, suggesting that future studies should incorporate wearable technology for more accurate monitoring. Larger, long-term studies are needed to confirm these results and evaluate the sustained effects of telerehabilitation on physical performance and QoL. Additionally, while the program offered varying exercise levels, further tailoring based on individual factors such as disease severity and comorbidities may improve outcomes.

In conclusion, telerehabilitation via the Health Village platform was a feasible and effective way to enhance functional capacity in patients with pulmonary fibrosis, including those with IPF and NSIP. The observed improvements in physical performance supported telerehabilitation as a practical alternative or complement to traditional programs, particularly for patients with limited mobility or those living in remote areas. Despite certain limitations, the findings provided a valuable foundation for future research. As telerehabilitation continues to evolve, it offers promising opportunities to expand access to care and improve outcomes and QoL for individuals with chronic ILDs.

## Abbreviations Used

6MWT: 6-min walk test
BMI: Body mass index
DG: Diagnosis
DLCO: Diffusion capacity of the lung for carbon monoxide
EU: European Union
FEV1: Forced expiratory volume in 1 sec
FVC: Forced vital capacity
ILD: Interstitial lung diseases
IPF: Idiopathic pulmonary fibrosis
MMRC: Modified Medical Research Council dyspnea scale
NSIP: Nonspecific interstitial pneumonia
O_2_: Qxygen
QoL: Quality of life
RAND-36: 36-item Short Form Survey for health-related quality of life
SD: Standard deviation
VAS: Visual analog scale
